# Impact of the response to platinum-based chemotherapy on the second-line immune checkpoint inhibitor monotherapy in non-small cell lung cancer with PD-L1 expression ≤49%: a multicenter retrospective study

**DOI:** 10.3389/fonc.2024.1303543

**Published:** 2024-01-26

**Authors:** Akihiro Yoshimura, Takayuki Takeda, Nobutaka Kataoka, Keiko Tanimura, Mototaka Fukui, Yusuke Chihara, Shota Takei, Hayato Kawachi, Kentaro Nakanishi, Yuta Yamanaka, Nobuyo Tamiya, Ryoichi Honda, Naoko Okura, Takahiro Yamada, Kiyoaki Uryu, Junji Murai, Shinsuke Shiotsu, Hiroshige Yoshioka, Tadaaki Yamada, Takayasu Kurata, Koichi Takayama

**Affiliations:** ^1^ Department of Respiratory Medicine, Japanese Red Cross Kyoto Daini Hospital, Kyoto, Japan; ^2^ Department of Pulmonary Medicine, Graduate School of Medical Science, Kyoto Prefectural University of Medicine, Kyoto, Japan; ^3^ Department of Respiratory Medicine, Uji-Tokushukai Medical Center, Uji, Kyoto, Japan; ^4^ Department of Thoracic Oncology, Kansai Medical University Hospital, Hirakata, Osaka, Japan; ^5^ Department of Respiratory Medicine, Rakuwakai Otowa Hospital, Kyoto, Japan; ^6^ Department of Respiratory Medicine, Asahi General Hospital, Asahi, Chiba, Japan; ^7^ Department of Pulmonary Medicine, Matsushita Memorial Hospital, Moriguchi, Osaka, Japan; ^8^ Department of Respiratory Medicine, Yao Tokushukai General Hospital, Yao, Osaka, Japan; ^9^ Department of Respiratory Medicine, Japanese Red Cross Kyoto Daiichi Hospital, Kyoto, Japan

**Keywords:** immune checkpoint inhibitor monotherapy, modified Glasgow prognostic score, non-small cell lung cancer, platinum-based chemotherapy, predictive marker

## Abstract

**Introduction:**

The efficacy of second-line immune checkpoint inhibitor (ICI) therapy is limited in non-small cell lung cancer (NSCLC) patients with ≤ 49% PD-L1 expression. Although chemoimmunotherapy is a promising strategy, platinum-based chemotherapy followed by ICI monotherapy is often used to avoid synergistic adverse events. However, predictors of the efficacy of ICI monotherapy after platinum-based chemotherapy in NSCLC with ≤ 49% PD-L1 expression remain scarce.

**Methods:**

This multicenter retrospective study evaluated 54 advanced or recurrent NSCLC patients with ≤ 49% PD-L1 expression who were treated with second-line ICI monotherapy following disease progression on first-line platinum-based chemotherapy at nine hospitals in Japan. The impact of response to platinum-based chemotherapy on the efficacy of subsequent ICI monotherapy was investigated.

**Results:**

The response to first-line platinum-based chemotherapy was divided into two groups: the non-progressive disease (PD) group, which included patients who did not experience disease progression after four cycles of chemotherapy, and the PD group, which included patients who showed initial PD or could not maintain disease control during the four cycles of chemotherapy and switched to second-line ICI monotherapy. Among the 54 patients, 32 and 22 were classified into the non-PD and PD groups, respectively. The non-PD group showed better response rates (p = 0.038) and longer overall survival (OS) with ICI monotherapy (p = 0.023) than the PD group. Multivariate analysis identified that maintaining a non-PD status after four cycles of chemotherapy was an independent prognostic factor for ICI monotherapy (p = 0.046). Moreover, patients with a modified Glasgow Prognostic Score (mGPS) of 0 showed a tendency for longer OS with ICI monotherapy (p = 0.079), and there was a significant correlation between maintaining non-PD after four cycles of chemotherapy and an mGPS of 0 (p = 0.045).

**Conclusion:**

Maintaining a non-PD status after four cycles of platinum-based chemotherapy was a predictor of OS after second-line ICI monotherapy. These findings will help physicians select the most suitable treatment option for NSCLC patients who were treated with platinum-based chemotherapy and switched to second-line treatment. Those who experienced early PD during platinum-based chemotherapy should not be treated with ICI monotherapy in the second-line setting.

## 1 Introduction

Lung cancer is the most common cause of cancer-related death worldwide (1), with non-small cell lung cancer (NSCLC) comprising approximately 85% of cases (2). The introduction of immune checkpoint inhibitors (ICIs) has dramatically altered treatment strategies for several cancers, including melanoma, lung cancer, and renal cell carcinoma (3). Programmed death-ligand 1 (PD-L1) expression in tumor cells serves as a positive predictive biomarker during ICI treatment in patients with advanced NSCLC (4). This is attributable to the fact that increased PD-L1 expression in tumor cells suppresses T-cell activation and proliferation by inducing effector T-cell apoptosis, resulting in an escape from immune responses (5, 6).

In the first-line setting, ICI monotherapy does not provide longer overall survival (OS) than platinum-based chemotherapy in patients with advanced NSCLC with low (1–49%) PD-L1 expression (7–9) compared to those with high (≥ 50%) PD-L1 expression on tumor cells (4). In contrast, chemoimmunotherapy (CIT) demonstrated superiority in OS over platinum-based chemotherapy as the first-line treatment for NSCLC, irrespective of PD-L1 expression status (10–13). Although an increase in adverse events associated with first-line CIT was shown in a network meta-analysis of 16 randomized controlled trials (14), CIT was adopted in patients with low or negative PD-L1 expression and a low rate of treatment failure. In contrast, sequential administration of first-line platinum-based chemotherapy followed by ICI monotherapy is sometimes selected to avoid the synergistic adverse events of CIT. This strategy is based on phase III trials that demonstrated the superiority of second-line ICI monotherapy over docetaxel (15–18).

CD8-positive tumor-infiltrating lymphocytes (TILs), which are representative markers of the tumor microenvironment (TME), also serve as predictors of anti-programmed cell death-1 (PD-1) treatment in NSCLC (19). CD8-positive TILs are known to increase after neoadjuvant chemotherapy in resected NSCLC specimens, suggesting that cytotoxic chemotherapy promotes antitumor immunity through T- and B-cell recruitment in the immune microenvironment (20). CD8-positive TILs are significantly increased in patients with advanced gastric cancer who respond to cytotoxic chemotherapy compared to those who do not (21). Thus, the response to first-lineplatinum-based chemotherapy in advanced NSCLC with PD-L1 expression of ≤49% could affect the efficacy of second-line ICI monotherapy; however, this has never been investigated.

In addition to the TME, cancer cachexia is an important host condition that affects the response to tumor cells (22). The modified Glasgow Prognostic Score (mGPS) is defined by serum C-reactive protein (CRP) and albumin levels (23, 24). Cancer cachexia can be assessed by mGPS which focuses on nutrition and systemic inflammation (25). Since neutrophil and platelet are known to have pro-inflammatory role in patients with cancer, while lymphocyte lead to tumor suppression, the neutrophil-to-lymphocyte ratio (NLR) and the platelet-to-lymphocyte ratio (PLR) are considered as useful immunological and nutritional markers in predicting the outcomes (26, 27).

In this multicenter retrospective study, the impact of response to platinum-based chemotherapy on the efficacy of subsequent ICI monotherapy was investigated. The differences among the subgroups with PD-L1 expression of 1–49% and <1% and the influence of mGPS values, NLR, and PLR on OS were also evaluated.

## 2 Patients and methods

### 2.1 Study population

We analyzed the electronic medical records of consecutive patients with advanced or recurrent NSCLC with PD-L1 expression ≤49% between January 1, 2016, and September 30, 2021, at nine hospitals in Japan. The study protocol was approved by the Ethics Committees of the Japanese Red Cross Kyoto Daini Hospital (February 2, 2022; S2021-43) and each participating hospital. The requirement for consent was waived due to the retrospective nature of the study and its anonymity. Patients were allowed to withdraw their data and relevant information, which were available on each hospital’s website.

Inclusion criteria were as follows: (a) patients aged 20 years or older; (b) those with pathologically diagnosed NSCLC without driver gene alteration; (c) those with metastatic NSCLC or NSCLC with postoperative recurrence; (d) PD-L1 expression on tumor cells ≤49%; e) patients with evaluable lesions by the Response Evaluation Criteria in Solid Tumors (RECIST) version 1.1.; (f) patients treated with first-line platinum-based chemotherapy followed by second-line ICI monotherapy during the study period. Adjuvant chemotherapy after surgery was not considered platinum-based chemotherapy.

### 2.2 Data collection

The following clinical data were obtained from electronic medical records: age, sex, smoking status, Eastern Cooperative Oncology Group performance status (ECOG-PS), clinical stage, histological subtype, PD-L1 expression in tumor cells, and pretreatment serum CRP and albumin levels at the time of ICI monotherapy administration. Patients with missing data were excluded from the analysis.

### 2.3 Clinical outcomes

Either computed tomography scan or magnetic resonance imaging was performed to determine complete response (CR), partial response (PR), stable disease (SD), progressive disease (PD), and not evaluable (NE) status based on the RECIST version 1.1. Objective response rate (ORR) and disease control rate (DCR) were defined as “the percentage of patients in the study or treatment group who achieved CR or PR after the treatment” and “the percentage of patients in the study or treatment group who achieved CR, PR, and SD”, respectively (28). Progression-free survival (PFS) was defined as the duration from the initiation of ICI monotherapy to the date of disease progression or death, whichever came first. Patients who remained alive without disease progression were censored at the date of their last imaging examination. OS was defined as the duration from the initiation of ICI monotherapy to death. Patients who were still alive at the time of data acquisition were censored at the date of the last visit.

### 2.4 PD-L1 testing

PD-L1 expression was evaluated in pretreatment samples by PD-L1 immunohistochemistry (IHC) using the 22C3 pharmDx assay (Dako North America, USA). Patients were categorized into two groups based on their PD-L1 expression status: low (1–49%) and negative (< 1%).

### 2.5 Modified Glasgow prognostic score

The mGPS was determined as previously described (24). Patients with neither elevated CRP levels (> 1 mg/dl) nor hypoalbuminemia (< 3.5 g/dl) were assigned a score of 0; those with either of these biochemical abnormalities were assigned a score of 1; and those with both abnormalities were assigned a score of 2.

### 2.6 Neutrophil-to-lymphocyte ratio and platelet-to-lymphocyte ratio

NLR was the ratio of absolute neutrophil count (/µL) divided by absolute lymphocyte count (/µL). PLR was the ratio of absolute platelet count (/µL) divided by absolute lymphocyte count. Based on the previous reports (26, 27), the cut-off values for NLR and PLR were set at < 3.5 or ≥ 3.5 and < 200 or ≥ 200, respectively.

### 2.7 Statistical analysis

PFS and OS curves were plotted using the Kaplan–Meier method. The log-rank test was used to evaluate the PFS and OS. The hazard ratios (HRs) for PFS and OS were determined using a univariate Cox proportional hazards model. Cox proportional hazard models were used to evaluate the patients’ background factors. To construct the multivariate model, we selected factors associated with OS that were most relevant to the univariate analysis results and previous reports. All statistical analyses were performed using the GraphPad Prism software (v.9.41; GraphPad Software, San Diego, CA, USA). Statistical significance was set at p < 0.05.

## 3 Results

### 3.1 Characteristics of patients before immune checkpoint inhibitor monotherapy

Among the 54 patients enrolled in this study with advanced or postoperative recurrent NSCLC with low (1–49%) or negative (< 1%) PD-L1 expression, the median age was 72.5 years (range: 33.0–85.0). Of these patients, 49 (90.7%) were males, 49 (90.7%) were current or former smokers, and all (100.0%) had an ECOG-PS of 0 or 1 (**Table 1**). Nine patients (16.7%) experienced postoperative recurrence, with adenocarcinoma being the most prevalent type (55.6%). PD-L1 expression in tumor cells was low in 43 patients and negative in 11 patients.

**Table 1:** Patients characteristics.

		n = 54
Median age, years (range)		72.5 (33.0–85.0)
Age categorization, n (%)	<75	36 (66.7)
	≥75	18 (33.3)
Sex, n (%)	Male	49 (90.7)
	Female	5 ( 9.3)
Smoking status, n (%)	Current or former	49 (90.7)
	Never	5 ( 9.3)
PS, n (%)	0	9 (16.7)
	1	45 (83.3)
Disease stage, n (%)	III	4 ( 7.4)
	IV	41 (75.9)
	Postoperative relapse	9 (16.7)
Histology, n (%)	Adenocarcinoma	30 (55.6)
	Others	24 (44.4)
PD-L1 TPS, n (%)	≥50%	0 ( 0.0)
	1-49%	43 (79.6)
	<1%	11 (20.4)
Response of platinum doublet, n (%)	PR	23 (42.6)
	SD	22 (40.7)
	PD	9 (16.7)
	NE	0 ( 0.0)
	ORR (95% CI)	42.6% (29.2–56.8%)
	DCR (95% CI)	83.3% (70.7–92.1%)
Disease control after 4 cycles of platinum doublet, n (%)	Achieved	32 (59.3)
	Not achieved	22 (40.7)
Response of ICIs monotherapy, n (%)	PR	4 ( 7.4)
	SD	18 (33.3)
	PD	29 (53.7)
	NE	3 ( 5.6)
	ORR (95% CI)	7.8% (2.2–18.9%)
	DCR (95% CI)	43.1% (29.3–57.8%)

PS, performance status; PR, partial response; SD, stable disease; PD, progressive disease; NE, not evaluable; ORR, objective response rate; CI, confidence interval; DCR, disease control rate; PD-L1, programmed death-ligand 1; TPS, tumor proportion score; ICI, immune-checkpoint inhibitor.

The objective responses to the first-line platinum-based chemotherapy were as follows: CR in 0, PR in 23 (42.6%), SD in 22 (40.7%), PD in nine (16.7%), and NE in no patients. ORR was 42.6% (95% confidence interval [CI]: 29.2-56.8) and DCR was 83.3% (95% CI: 70.7-92.1).

### 3.2 Relationship between the response to platinum-based chemotherapy and clinicopathological features

The patients were divided into two groups based on the response to first-line platinum-based chemotherapy: the non-PD group, which included patients who did not experience disease progression after four cycles of induction chemotherapy, and the PD group, which included patients who showed initial PD or could not maintain disease control during the four cycles of induction chemotherapy and switched to second-line ICI monotherapy. Among the 54 patients, 32 and 22 were classified into the non-PD and PD groups, respectively (**Supplementary Table 1**). There was no significant difference between the two groups in terms of clinicopathological features, except for adenocarcinoma histology, which showed better disease control than non-adenocarcinoma (p = 0.027). Among the 32 patients in the non-PD group, 25 and seven patients had low and negative PD-L1 expression, respectively. Among the 22 patients in the PD group, 18 and four patients had low and negative PD-L1 expression, respectively (**Supplementary Table 1**).

### 3.3 Significance of the response to platinum-based chemotherapy and the efficacy of immune checkpoint inhibitor monotherapy

**Figure 1:**
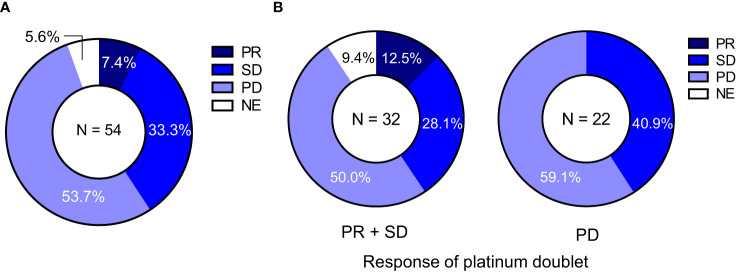
Second-line ICI monotherapy efficacy according to the response to the first-line platinum-based chemotherapy. **(A)** The response to second-line ICI monotherapy in 54 patients with programmed death ligand 1 (PD-L1) expression ≤49%. **(B)** The response to second-line ICI monotherapy in patients with NSCLC and PD-L1 expression ≤49% stratified according to the response (non-PD vs. PD) to the first-line platinum-based chemotherapy. There was a significant relationship in ORR of second-line ICI monotherapy between the response (non-PD and PD) to the first-line platinum-based chemotherapy (13.8% vs. 0.0%, p = 0.038). ICI, immune checkpoint inhibitor; NSCLC, non-small cell lung cancer; PD-L1, programmed death-ligand 1; ORR, objective response rate; PD, progressive disease; PR, partial response; SD, stable disease.

Among 54 patients, the objective responses to the second-line ICI monotherapy were as follows: CR in 0, PR in 4 (7.4%), SD in 18 (33.3%), PD in 29 (53.7%), and NE in 3 (5.6%) (**Table 1**). The ORR and DCR of the second-line ICI monotherapy were 7.8% (95% CI:2.2-18.9) and 43.1% (95% CI:29.3-57.8), respectively (**Table 1**; **Figure 1A**), showing lower ORR and DCR compared to those of platinum-based chemotherapy (42.6% and 83.3%, respectively) (**Table 1**).

The effect of the response to platinum-based chemotherapy on the efficacy of ICI monotherapy was evaluated. The ORR for ICI monotherapy was significantly higher in the non-PD group than in the PD group (13.8% vs. 0.0%, p = 0.038) (**Figure 1B**).

### 3.4 Predictor for the progression-free and overall survival of immune checkpoint inhibitor monotherapy

**Figure 2:**
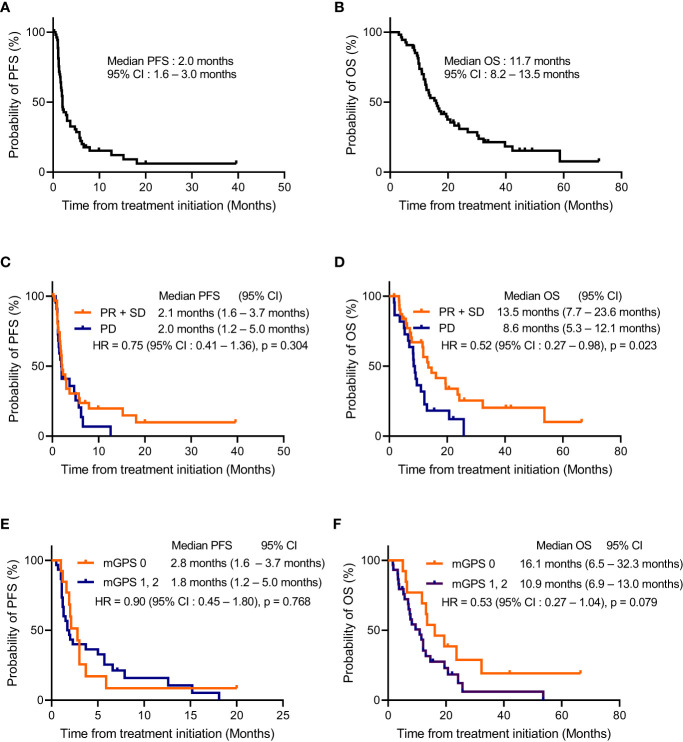
Kaplan-Meier estimates of progression-free survival and overall survival of second-line immune checkpoint inhibitor monotherapy. Kaplan–Meier estimates for progression-free survival [PFS: **(A)**] and overall survival [OS: **(B)**] in patients receiving immune checkpoint inhibitor (ICI) monotherapy after disease progression on platinum-based chemotherapy (n = 54). Kaplan–Meier estimates for PFS **(C)** and OS **(D)** of second-line ICI monotherapy were classified according to the response to first-line platinum-based chemotherapy (non-progressive disease [PD] vs. PD). The median PFS was 2.1 months in the non-PD group (95% confidence interval [CI]: 1.6–3.7 months) and 2.0 months in the PD group (95% CI: 1.2–5.0 months) with a p-value = 0.304, and the median OS was 13.5 months in the non-PD group (95% CI:7.7–23.6 months) and 8.6 months in the PD group (95% CI:5.3–12.1 months) with a p-value = 0.023. Kaplan–Meier estimates for PFS **(E)** and OS **(F)** of second-line ICI monotherapy were classified using the modified Glasgow Prognostic Score (mGPS; 0 vs. 1–2). The median PFS of ICIs monotherapy was 2.8 months in the subgroup with mGPS of 0 (95% CI: 1.6–3.7 months) and 1.8 months in the subgroup with mGPS of 1–2 (95% CI: 1.2–5.0 months) with a p-value = 0.768, and the median OS was 16.1 months in the subgroup with mGPS of 0 (95% CI: 6.5–32.3 months) and 10.9 months in the subgroup with mGPS of 1–2 (95% CI: 6.9–13.0 months) with a p-value = 0.079. PFS, progression-free survival; OS, overall survival; NSCLC, non-small cell lung cancer; PD-L1, programmed death-ligand 1; ICI, immune checkpoint inhibitor; PR, partial response; SD, stable disease; PD, progression disease; CI, confidence interval; mGPS, modified Glasgow Prognostic Score; NE, not evaluable.

Subsequently, the predictors of PFS and OS of second-line ICI monotherapy for NSCLC with PD-L1 expression ≤49% were investigated. The median follow-up period was 11.0 months (range: 1.6–66.5). The median PFS and OS of ICI monotherapy were 2.0 months (95% CI: 1.6–3.0) and 11.7 months (95% CI: 8.2–13.5), respectively (**Figures 2A, B**).

Univariate analysis identified non-PD group (maintaining disease control after 4 cycles of first-line platinum-based chemotherapy) as a predictor for longer OS with ICI monotherapy; median OS in the non-PD group (13.5 months [95% CI, 7.7–23.6]) and in the PD group (8.6 months [95% CI, 5.3–12.1]) (p = 0.023) (**Table 2**; **Figure 2D**). In contrast, there was no significant difference in the PFS between the non-PD and PD groups (P = 0.304) (**Figure 2C**). There was no significant difference between tumor PD-L1 expression of 1–49% and that of <1% in PFS (p = 0.441) and OS (p = 0.485) (**Table 2**).

**Table 2:** Cox proportional hazard models for PFS and OS in patients with non-small cell lung cancer who received ICIs monotherapy, univariate analysis.

Characteristics		Patient’s No.	Median PFS (95% CI), months	*P* value	Median OS (95% CI), months	*P* value
Age categorization	<75	36	2 (1.5–3.7)	0.975	11.5 (7.1–16.1)	0.784
	≥75	18	2.3 (1.2–6.2)		11.7 (6.1–13.5)	
Sex	Male	49	2.0 (1.6–3.7)	0.413	11.7 (8.2–13.5)	0.849
	Female	5	1.9 (1.1–NE)		10.9 (5.3–NE)	
Smoking status	Current or former smoker	5	1.9 (1.0–NE)	0.246	7.7 (3.8–NE)	0.728
	Never smoker	49	2.0 (1.6–3.7)		11.7 (8.3–13.5)	
PS	0	9	2.0 (1.0–4.7)	0.870	19.4 (3.8–NE)	0.200
	1	45	2.0 (1.5–3.7)		11.5 (7.5–13.1)	
Disease stage	III	4	3.6 (2.1–NE)	0.903	12.4 (9.5–NE)	0.502
	IV	41	2.0 (1.3–3.7)		8.8 (6.5–13.5)	
	Postoperative relapse	9	1.9 (1.1–15.2)		16.1 (7.7–32.3)	
Histology	Adenocarcinoma	30	2.0 (1.3–5.0)	0.772	13.5 (7.7–20.7)	0.211
	Others	24	2.0 (1.5–3.7)		9.0 (6.9–11.7)	
PD-L1 TPS	1–49%	43	2.0 (1.6–3.0)	0.441	10.9 (7.5–12.1)	0.485
	< 1%	11	2.1 (1.1–7.9)		14.6 (3.4–NE)	
Disease control after 4 cycles of platinum-based doublet chemotherapy	Achieved	32	2.1 (1.6–3.7)	0.304	13.5 (7.7–23.6)	0.023
	Not achieved	22	2.0 (1.2–5.0)		8.6 (5.3–12.1)	
Modified Glasgow Prognositc Score	0	13	2.8 (1.6–3.7)	0.768	16.1 (6.5–32.3)	0.079
	1, 2	30	1.8 (1.2–5.0)		10.9 (6.9–13.0)	
Neutrophil-to-lymphocyte ratio	<3.5	24	3.0 (1.7–5.0)	0.143	13.0 (9.5–23.6)	0.145
	≥3.5	20	1.4 (1.1–2.1)		7.1 (3.5–13.1)	
Platelet-to-lymphocyte ratio	<200	30	2.0 (1.6–3.0)	0.596	12.1 (7.1–19.4)	0.139
	≥200	14	1.7 (1.1–5.7)		9.5 (4.4–13.5)	

PFS, progression-free survival; OS, overall survival; ICI, immune-checkpoint inhibitor; CI, confidence interval; PS, performance status; NE, not evaluable; PD-L1, programmed death-ligand 1; TPS, tumor proportion score.

Multivariate analysis showed that the non-PD group was an independent predictor for OS of ICI monotherapy (HR: 0.49, 95% CI: 0.24–0.99, p = 0.046) (**Table 3**).

**Table 3:** Cox proportional hazard models for OS in patients with non-small cell lung cancer who received ICIs monotherapy, multivariate analysis.

Items	Hazard ratio (95% CI)	*P* value
Age ≥ 75	0.81 (0.40-1.65)	0.560
Adenocarcinoma	0.83 (0.43–1.59)	0.580
Achievement of disease control after 4 cycles of platinum-based doublet chemotherapy	0.49 (0.24-0.99)	0.046

OS, overall survival; ICI, immune-checkpoint inhibitor; CI, confidence interval.

Furthermore, in NSCLC with PD-L1 expression of 1–49%, the median OS of ICI monotherapy was significantly longer in the non-PD group (13.5 months [95% CI, 7.5–24.2]) than in the PD group (8.3 months [95% CI, 5.1–9.5]), with a p-value of 0.003 (**Supplementary Figure 1B**), while there was no significant difference in the PFS between the two groups (p = 0.473) (**Supplementary Figure 1A**). However, there was no significant difference in PFS (p = 0.519) and OS (p = 0.555) based on PD-L1 expression in the < 1% subgroup between the non-PD and PD groups (**Supplementary Figures 1C, D**).

### 3.5 Influence of immunological and nutritional markers and the response to platinum-based chemotherapy on the efficacy of immune checkpoint inhibitor monotherapy

Serum CRP and albumin levels were available at the start of ICI monotherapy in 43 patients, among whom 13, 16, and 14 patients were categorized as having an mGPS of 0, 1, and 2, respectively. Neutrophil, lymphocyte, and platelet counts at the start of ICI monotherapy were available among 44 patients. Among 44 patients, 20 and 24 patients showed NLR < 3.5 and ≥ 3.5 and < 3.5, while 14 and 30 patients showed PLR < 200 and ≥ 200, respectively. The relationship between the response to first-line platinum-based chemotherapy and the mGPS, NLR and PLR values at the start of ICI monotherapy was assessed in 43 patients. Although there was no significant difference between the effect of platinum-based chemotherapy and the NLR or PLR values, patients with an mGPS score of 0 were significantly more prevalent in the non-PD group, which maintained disease control after four cycles of induction chemotherapy (42.3%), compared to the PD group (11.8%), with a p-value = 0.045 (**Supplementary Table 2**). In contrast to the NLR and PLR showing no significant difference in PFS and OS (**Supplementary Figure 2**), the median OS of ICI monotherapy was relatively longer in patients with mGPS of 0 (16.1 months [95% CI: 6.5–32.3]) than in patients with mGPS of 1–2 (10.9 months [95% CI: 6.9–13.0]), with a p-value = 0.079 (**Table 2**; **Figure 2F**). In contrast, there was no significant difference in the PFS after ICIs monotherapy between patients with an mGPS of 0 and those with an mGPS of 1–2 (p = 0.768) (**Table 2**; **Figure 2E**).

## 4 Discussion

This study elucidated the impact of the response to first-line platinum-based chemotherapy on the efficacy of second-line ICI monotherapy for NSCLC with low or negative PD-L1 expression. The maintenance of non-PD after four cycles of platinum-based chemotherapy showed a strong relationship with the longer OS associated with subsequent ICI monotherapy for patients with NSCLC with PD-L1 expression of 1–49%. In contrast, this phenomenon was not observed in patients with NSCLC and PD-L1 expression <1%.

The median OS of the second-line ICI monotherapy among the subgroup with PD-L1 expression 1–49% who experienced PD before 4 cycles of platinum-based chemotherapy in this study (8.6 months) was shorter than that of the standard second-line treatment with docetaxel in a phase III trial in Japan (13.6 months) (29). ICI monotherapy was superior to docetaxel in phase III trials (15–18); therefore, identification of a population that would not benefit from ICI monotherapy is crucial. The results of this study suggest that patients who experience PD before 4 cycles of first-line platinum-based chemotherapy would not benefit from second-line ICI monotherapy, which would help physicians select docetaxel or nanoparticle albumin-bound (nab-) paclitaxel as the second-line treatment for this population (29).

In order to predict the responses to ICI-based treatment, monitoring quantified circulating cell-free DNA (cfDNA) is effective, which reflects longitudinal tumor dynamics in advance to the radiographic response (30). However, monitoring cfDNA has problem in its accessibility and cost.

In the current study, we aimed to find out the easily evaluable predictive makers. Thus, the relationship between mGPS and OS or PFS after ICI monotherapy was also investigated, considering the impact of cachexia, which is a poor prognostic factor for immunotherapy. A significant relationship was observed between the maintenance of disease control during the four cycles of platinum-based chemotherapy and the mGPS score at the start of ICI monotherapy (**Supplementary Table 2**). This is the first study to show the impact of disease control with first-line platinum-based chemotherapy on subsequent ICI monotherapy in patients with NSCLC with PD-L1 expression ≤49%. Although a significant correlation was not observed between mGPS at the start of ICI monotherapy and the median OS (**Table 2**), this finding suggests the significance of TME in ICI treatment.

The TME status is important for obtaining adequate effects from ICIs. Tumors with low or negative PD-L1 expression and scarce TILs are called “immune-desert” which are resistant to ICI monotherapy and need the activation of priming phase. In contrast, tumors with high PD-L1 expression and abundant TILs are called “immune-inflamed” which are sensitive to immunotherapy (31). To achieve the optimal “immune-inflamed” status by immunogenic cell death (32) and to obtain the most effective outcome, CIT was established as a new strategy in patients with NSCLC (15–18). Although CIT is effective compared to ICI monotherapy for NSCLC with PD-L1 expression ≤49%, the efficacy is not satisfactory compared to that with PD-L1 expression ≥50%. Furthermore, the increase in serious adverse events during CIT (14) is an obstacle in adopting CIT for NSCLC with PD-L1 expression ≤49%.

The median OS of CIT for NSCLC with PD-L1 expression ≤49% in updated 5-year follow-up of phase III trials remains at 15–21 months (33, 34). Since the OS of the non-PD group in the current study was comparable to that of the CIT group, the treatment strategy for NSCLC with PD-L1 expression ≤49% should be reconsidered.

Although NSCLC with low or negative PD-L1 expression is considered to show poor response to immunotherapy, the change in TME from “immune-desert” to “immune-inflamed” status with increased CD8-positive TILs prior to immunotherapy would lead to a good response to immunotherapy (31). An increase in CD8-positive TILs was observed in patients with resectable NSCLC who received neoadjuvant chemotherapy (20), showing the effect of platinum-based chemotherapy on the TME in NSCLC. When tumor cells are attacked by chemotherapy, the release of tumor-derived neoantigens into the blood facilitates the migration and functioning of antigen-presenting cells and augments antigen presentation, tumor recognition, and TIL activity (31, 35). The altered PD-L1 expression after neoadjuvant chemotherapy in patients with squamous NSCLC (36) should be also taken into account when treating patients with NSCLC with PD-L1 ≤49%, because underestimation of the expected outcome of ICI monotherapy in this population would lead to avoidance of the ICI treatment.

The TME status after disease progression with first-line chemotherapy should be re-evaluated to determine the most appropriate second-line treatment regimen; however, it is difficult to perform a re-biopsy and re-evaluate the immune status in all patients. Focusing on the impact of the TME on the development of cancer cachexia (37), immunological and nutritional indices such as mGPS, neutrophil-to-lymphocyte ratio, systemic immune-inflammation index, and platelet-to-lymphocyte ratio are surrogate markers in immunotherapy for NSCLC (38–41). However, there was only a slight correlation between mGPS and OS with ICI monotherapy in the current study, suggesting that mGPS is not an adequate predictor. In contrast, maintaining a non-PD status after four cycles of platinum-based chemotherapy was a predictor of the efficacy of second-line ICI monotherapy. Disease progression during the four cycles of induction chemotherapy indicates insufficient antitumor activity, failing to induce the activation of the priming phase, and failure to improve the TME for subsequent ICI monotherapy. The observed relationship between maintaining disease control and mGPS supports this speculation. This is consistent with the correlation between the prevalence of CD8-positive TILs and response to chemotherapy in advanced gastric cancer (21).

This study had several limitations. First, this retrospective study had a limited sample size and was susceptible to a selection bias. The enrollment of patients with advanced NSCLC with low or negative PD-L1 expression who were treated with platinum-based chemotherapy followed by ICI monotherapy was susceptible to bias. Second, all patients enrolled in this study were Japanese. Because the efficacy of the treatment for NSCLC has ethnic differences, this also led to bias. Thus, patients with a relatively favorable prognosis were included in this study. Despite these limitations, the novel findings of this study are useful for decision-making in patients with NSCLC with low or negative PD-L1 expression. Larger real-world clinical studies evaluating the predictive role of the response to first-line platinum-based chemotherapy are warranted.

## 5 Conclusion

Maintaining disease control (i.e., non-PD) after four cycles of platinum-based chemotherapy was a predictor of OS after second-line ICI monotherapy. These findings will help physicians select the most suitable treatment option for patients with NSCLC who were treated with platinum-based chemotherapy and subsequently with second-line treatment. Those who experienced early PD during platinum-based chemotherapy should not be treated with second-line ICI monotherapy, but with docetaxel or nab-paclitaxel. Further investigations are required to validate these findings.

## Data availability statement

The original contributions presented in the study are included in the article/**Supplementary Material**. Further inquiries can be directed to the corresponding author.

## Ethics statement

The studies involving humans were approved by the Ethics Committees of the Japanese Red Cross Kyoto Daini Hospital (February 2, 2022; S2021-43). The studies were conducted in accordance with the local legislation and institutional requirements. Written informed consent for participation was not required from the participants or the participants’ legal guardians/next of kin because of the retrospective study.

## Author contributions

AY: Conceptualization, Data curation, Formal analysis, Investigation, Resources, Writing – original draft. TT: Conceptualization, Data curation, Formal analysis, Writing – original draft, Writing – review & editing. NK: Data curation, Resources, Writing – review & editing. KKT: Data curation, Formal analysis, Validation, Writing – review & editing. MF: Conceptualization, Resources, Validation, Writing – review & editing. YC: Methodology, Resources, Validation, Writing – review & editing. ST: Data curation, Resources, Writing – review & editing. HK: Data curation, Resources, Validation, Writing – review & editing. KN: Data curation, Resources, Writing – review & editing. YY: Data curation, Resources, Validation, Writing – review & editing. NT: Resources, Validation, Writing – review & editing. RH: Conceptualization, Data curation, Resources, Validation, Writing – review & editing. NO: Data curation, Resources, Writing – review & editing. TY: Resources, Validation, Writing – review & editing. KU: Methodology, Resources, Validation, Writing – review & editing. JM: Data curation, Resources, Writing – review & editing. SS: Resources, Validation, Writing – review & editing. HY: Data curation, Validation, Writing – review & editing. TY: Data curation, Methodology, Validation, Writing – review & editing. TK: Formal analysis, Supervision, Validation, Visualization, Writing – review & editing. KCT: Formal analysis, Supervision, Validation, Visualization, Writing – review & editing.

## Supplementary material

Supplementary Figure 1 Kaplan-Meier estimates for progression-free survival and overall survival of second-line immune checkpoint inhibitor monotherapy according to programmed death-ligand 1 expression on tumor cells. Kaplan–Meier estimates for progression-free survival [PFS: **(A)**] and overall survival [OS: **(B)**] of immune checkpoint inhibitor (ICI) monotherapy in patients with programmed death-ligand 1 (PD-L1) expression of 1-49%, compared according to the response to first-line platinum-based chemotherapy (non-progressive disease [PD] vs. PD). The median PFS in the non-PD and PD subgroups were 2.0 months (95% confidence interval [CI]: 1.6–3.0 months) and 2.0 months (95% CI: 1.2–4.7 months), respectively (p = 0.473). The median OS in the non-PD and PD subgroups were 13.5 months (95% CI: 7.5–24.2 months) and 8.3 months (95% CI: 5.1–9.5 months), respectively (p = 0.003). Kaplan–Meier estimates for PFS **(C)** and OS **(D)** of ICI monotherapy in patients with PD-L1 expression <1%, compared according to the response to first-line platinum-based chemotherapy (non-PD vs. PD). The median PFS in the non-PD and PD subgroups were 2.1 months (95% CI: 1.0–18.1 months) and 3.7 months (95% CI: 1.4 months–not evaluable [NE]), respectively (p = 0.519). The median OS in the non-PD and PD subgroups were 14.6 months (95% CI: 3.3 months–NE) and 13.0 months (95% CI: 8.4 months–NE), respectively (p = 0.555). PFS, progression-free survival; OS, overall survival; PD-L1, programmed death-ligand 1; ICI, immune checkpoint inhibitor; PR, partial response; SD, stable disease; PD, progression disease; CI, confidence interval; NE, not evaluable.

Supplementary Figure 2 Kaplan-Meier estimates for progression-free survival and overall survival of second-line immune checkpoint inhibitor monotherapy according to neutrophil-to-lymphocyte ratio and platelet-to-lymphocyte ratio. Kaplan–Meier estimates for progression-free survival [PFS: **(A)**] and overall survival [OS: **(B)**] of immune checkpoint inhibitor (ICI) monotherapy according to neutrophil-to-lymphocyte ratio (NLR) (NLR < 3.5 vs. ≥ 3.5). The median PFS in patients with NLR of <3.5 and ≥3.5 were 3.0 months (95% confidence interval [CI]: 1.7–5.0 months) and 1.4 months (95% CI: 1.1–2.1 months), respectively (p = 0.143). The median OS in patients with NLR of < 3.5 and ≥ 3.5 were 13.0 months (95% CI: 9.5–23.6 months) and 7.1 months (95% CI: 3.5–13.1 months), respectively (p = 0.145). Kaplan–Meier estimates for PFS **(C)** and OS **(D)** of ICI monotherapy according to platelet-to-lymphocyte ratio (PLR) (PLR < 200 vs. ≥ 200). The median PFS in patients with PLR of < 200 and ≥ 200 were 2.0 months (95% CI: 1.6–3.0 months) and 1.7 months (95% CI: 1.1–5.7 months), respectively (p = 0.596). The median OS in patients with PLR of < 200 and ≥ 200 were 12.1 months (95% CI: 7.1–19.4 months) and 9.5 months (95% CI: 4.4–13.5 months), respectively (p = 0.139). PFS, progression-free survival; OS, overall survival; ICI, immune checkpoint inhibitor; NLR, neutrophil-to-lymphocyte ratio; CI, confidence interval; PLR, platelet-to-lymphocyte ratio.
