# Supplementary figures and images for: Impact of the response to platinum-based chemotherapy on the second-line immune checkpoint inhibitor monotherapy in non-small cell lung cancer with PD-L1 expression ≤49%: a multicenter retrospective study
Source: Front Oncol. 2024 Jan 26;14:1303543. doi: 10.3389/fonc.2024.1303543 (PMC10853418; doi:10.3389/fonc.2024.1303543)

## Slide 1
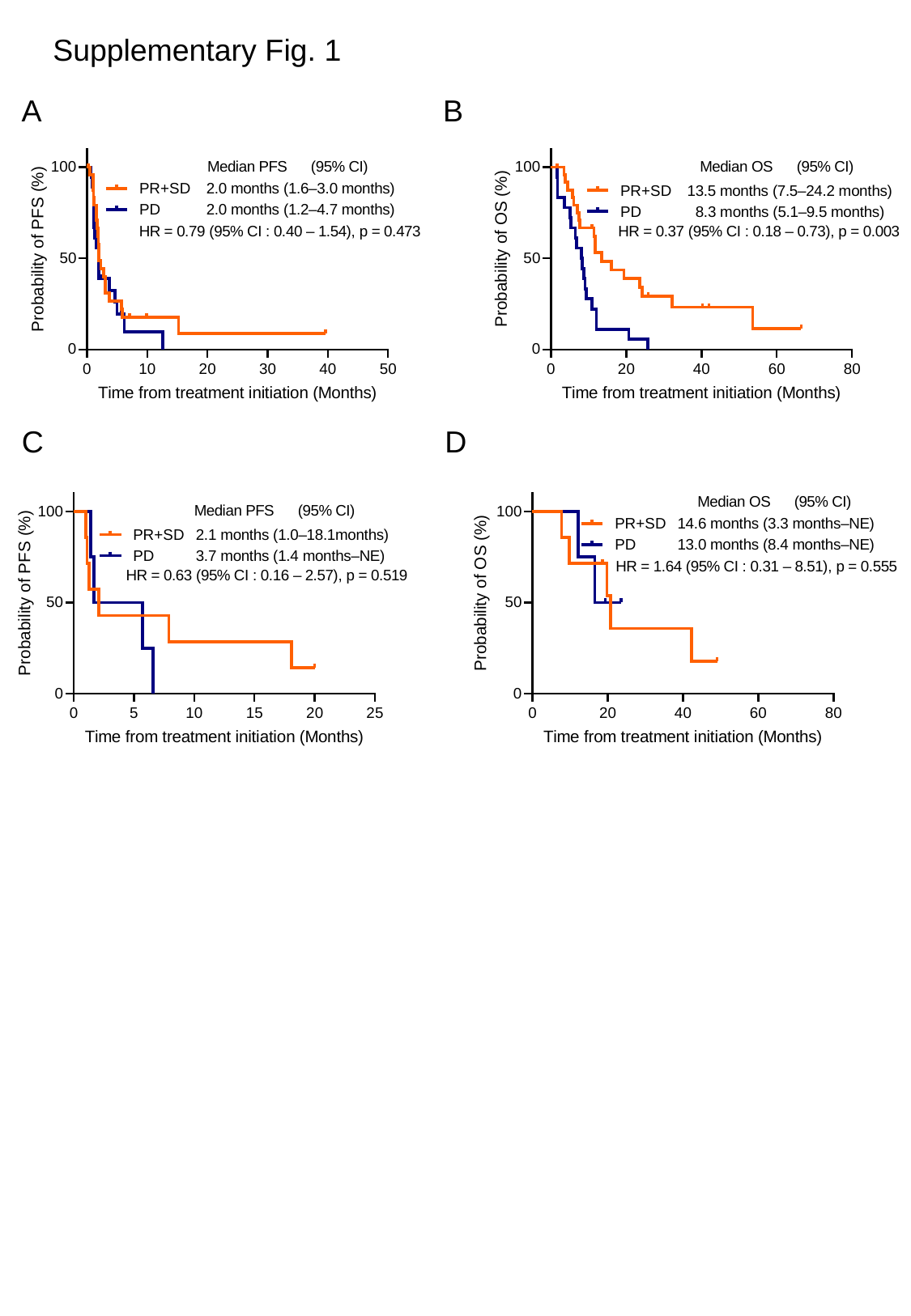

Supplementary Fig. 1
A
B
C
D

## Slide 2
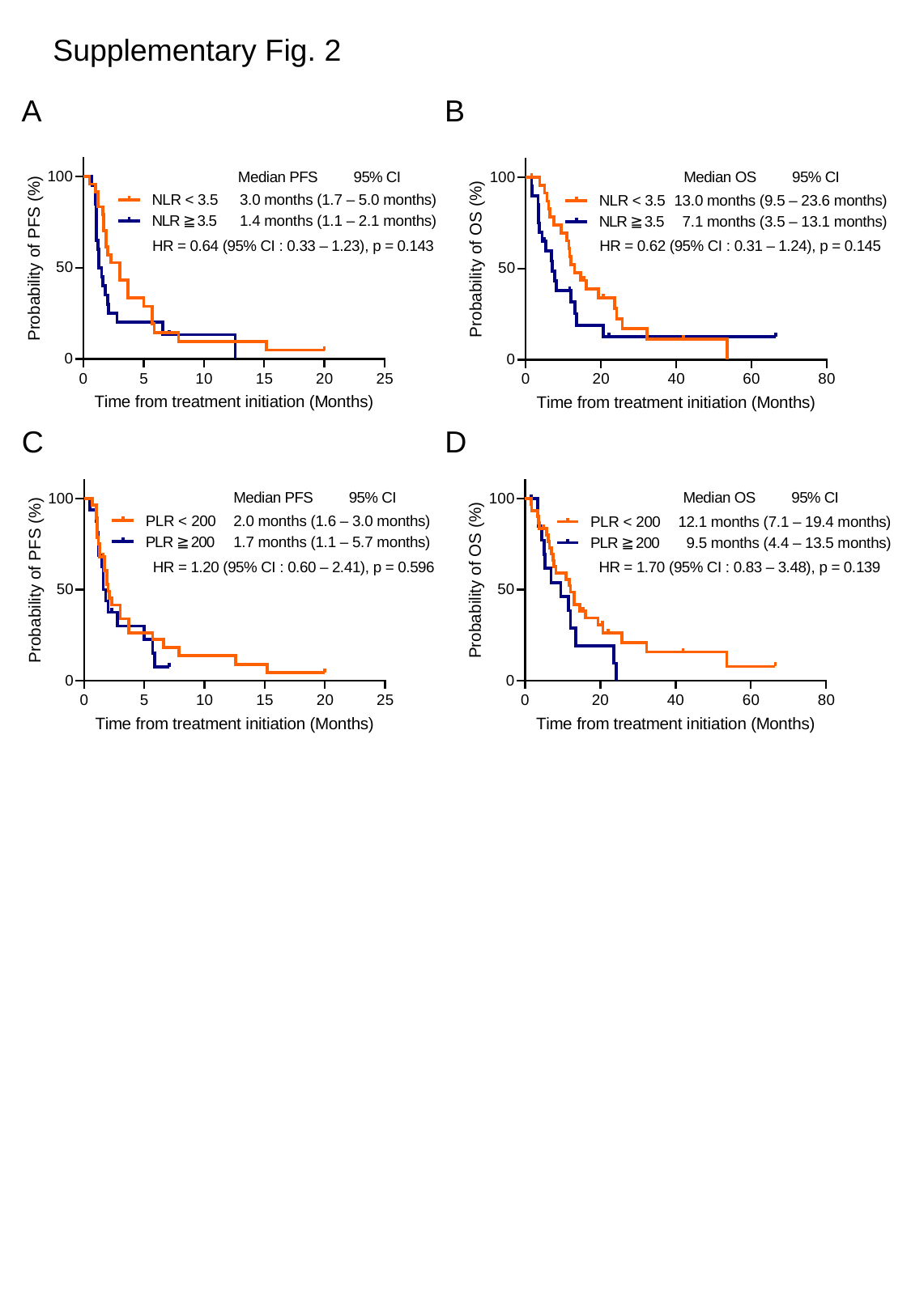

Supplementary Fig. 2
A
B
C
D

Supplement: Supplementary Figure 1 — Kaplan-Meier estimates for progression-free survival and overall survival of second-line immune checkpoint inhibitor monotherapy according to programmed death-ligand 1 expression on tumor cells. Kaplan–Meier estimates for progression-free survival [PFS: (A)] and overall survival [OS: (B)] of immune checkpoint inhibitor (ICI) monotherapy in patients with programmed death-ligand 1 (PD-L1) expression of 1-49%, compared according to the response to first-line platinum-based chemotherapy (non-progressive disease [PD] vs. PD). The median PFS in the non-PD and PD subgroups were 2.0 months (95% confidence interval [CI]: 1.6–3.0 months) and 2.0 months (95% CI: 1.2–4.7 months), respectively (p = 0.473). The median OS in the non-PD and PD subgroups were 13.5 months (95% CI: 7.5–24.2 months) and 8.3 months (95% CI: 5.1–9.5 months), respectively (p = 0.003). Kaplan–Meier estimates for PFS (C) and OS (D) of ICI monotherapy in patients with PD-L1 expression <1%, compared according to the response to first-line platinum-based chemotherapy (non-PD vs. PD). The median PFS in the non-PD and PD subgroups were 2.1 months (95% CI: 1.0–18.1 months) and 3.7 months (95% CI: 1.4 months–not evaluable [NE]), respectively (p = 0.519). The median OS in the non-PD and PD subgroups were 14.6 months (95% CI: 3.3 months–NE) and 13.0 months (95% CI: 8.4 months–NE), respectively (p = 0.555). PFS, progression-free survival; OS, overall survival; PD-L1, programmed death-ligand 1; ICI, immune checkpoint inhibitor; PR, partial response; SD, stable disease; PD, progression disease; CI, confidence interval; NE, not evaluable. [file Presentation_1.pptx]
